# Supplementary material for: Nuclear proteome analysis suggests spatial and temporal compartmentalization of metabolic enzymes during Trypanosoma cruzi cell cycle
Source: PLoS Negl Trop Dis. 2026 Sep 28;20(9):e0013299. doi: 10.1371/journal.pntd.0013299 (PMC13645223; doi:10.1371/journal.pntd.0013299)

Fig 1C - Coomassie-stained gel (SDS 15%). The gel was photographed using a smartphone camera, after electrophoresis and Coomassie staining. The image shown is the most complete available copy of the original photograph. Lane 1: Whole cell proteins extract. Lane 2: Nuclear proteins extract

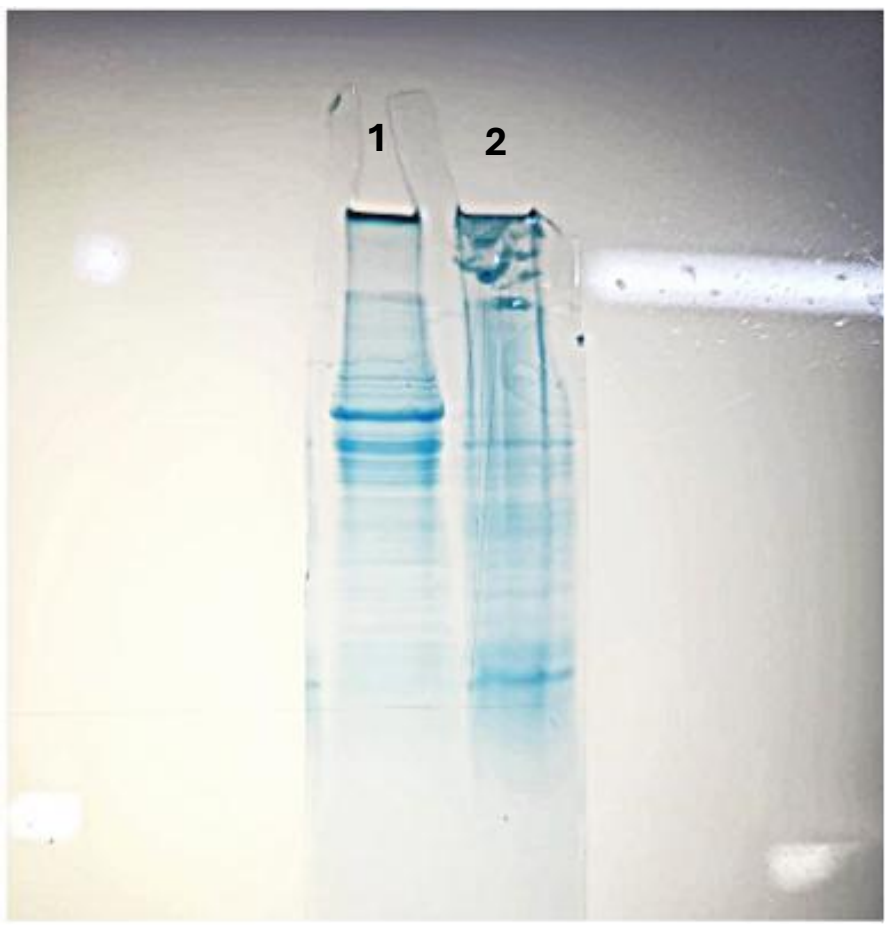

Fig 1D - Western blot analysis. Chemiluminescent signals were acquired using a UVITEC imaging system. The image shown is the most complete available copy corresponding to the published panel and was recovered from a previously cropped version of the original acquisition. The original uncropped image is no longer available because it was stored on the acquisition computer, which subsequently experienced a motherboard failure. Consequently, the molecular weight marker and any regions outside the displayed lanes are not available in the recovered image. Lane 1: Whole cell proteins extract. Lane 2: Nuclear proteins extract

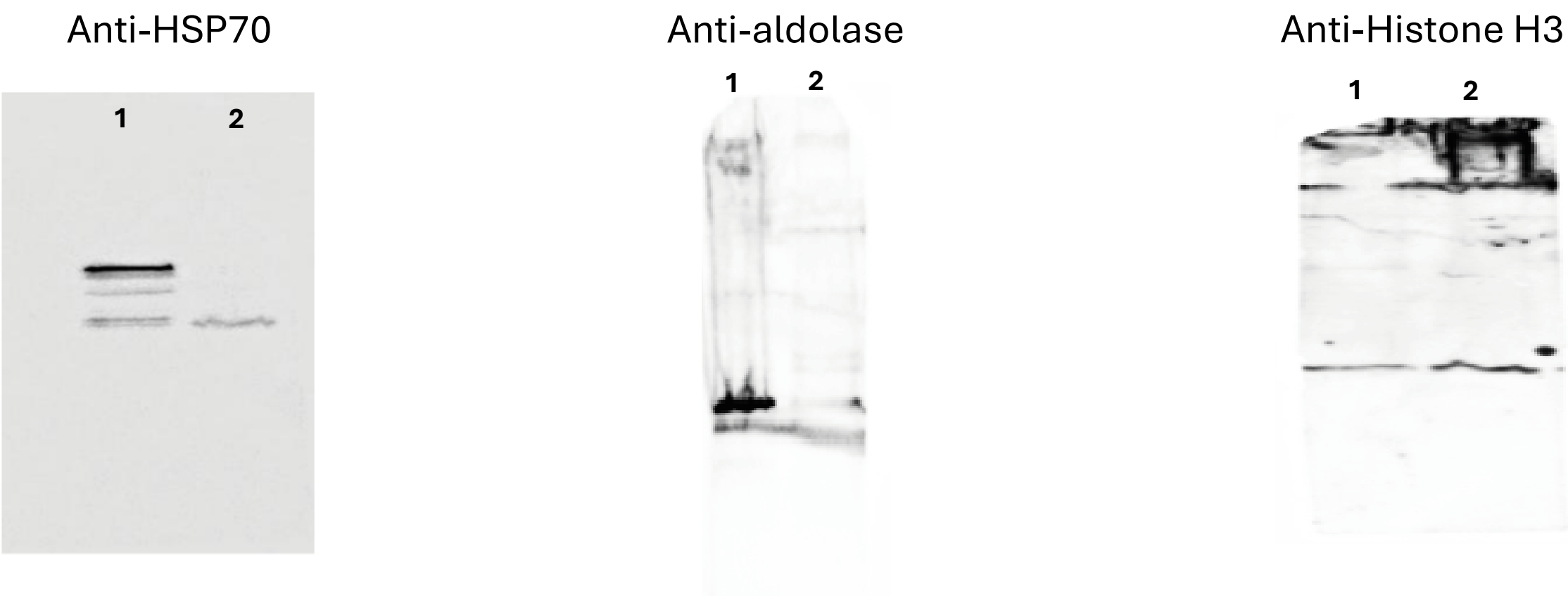

S4 Fig - Western blot validation of subcellular fractionation. Ponceau S staining of the membrane was photographed using a smartphone camera The molecular weight marker (PageRuler Prestained Protein Ladder, Thermo Scientific - 10–180 kDa) is visible in the

original membrane image. Chemiluminescent signals were acquired using a UVITEC imaging system.
Lane E: total cell extract; Lane C: cytoplasmic fraction; Lane N: nuclear fraction. H3 and eIF5a were analyzed on separate membranes and imaged independently using the UVITEC imaging system. Lanes marked with “X” were present in the original blot but were not included in the corresponding published figure.

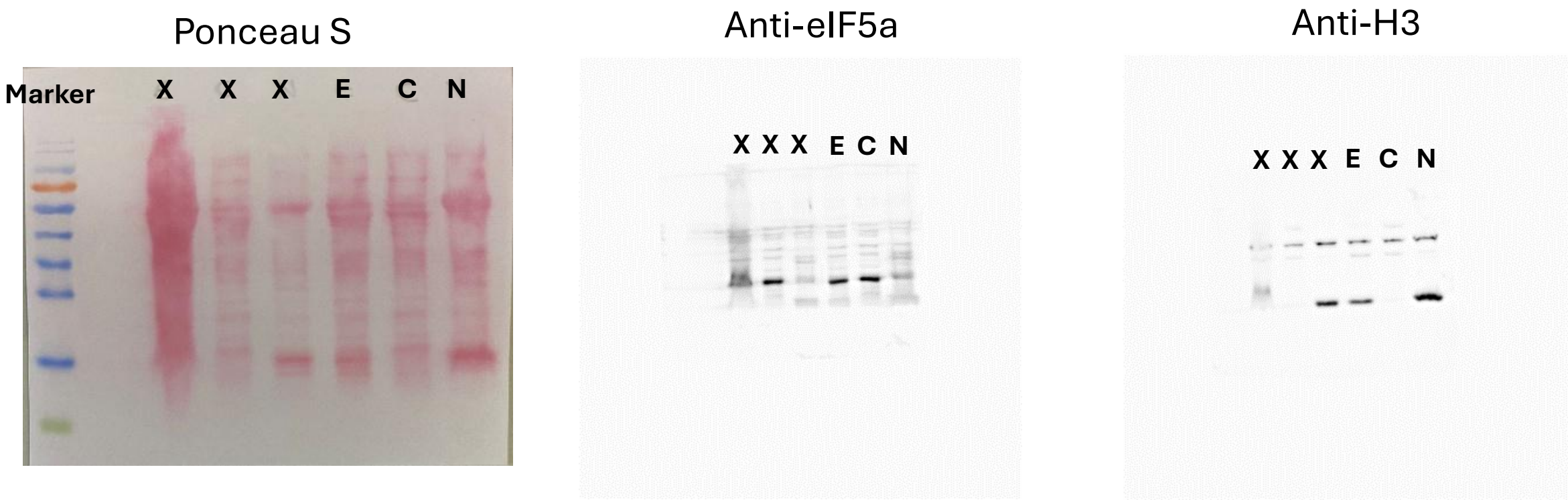

S5 Fig - Western blot analysis of Citrate Synthase (CS), Hexokinase (HK) and Glycerol kinase (GK). CS, HK, and GK were analyzed on separate membranes. The three membranes were placed together in the UVITEC imaging system and imaged simultaneously using the same exposure time and acquisition settings. A PageRuler Prestained Protein Ladder, 10–180 kDa (Thermo Scientific), was used as the molecular weight marker.

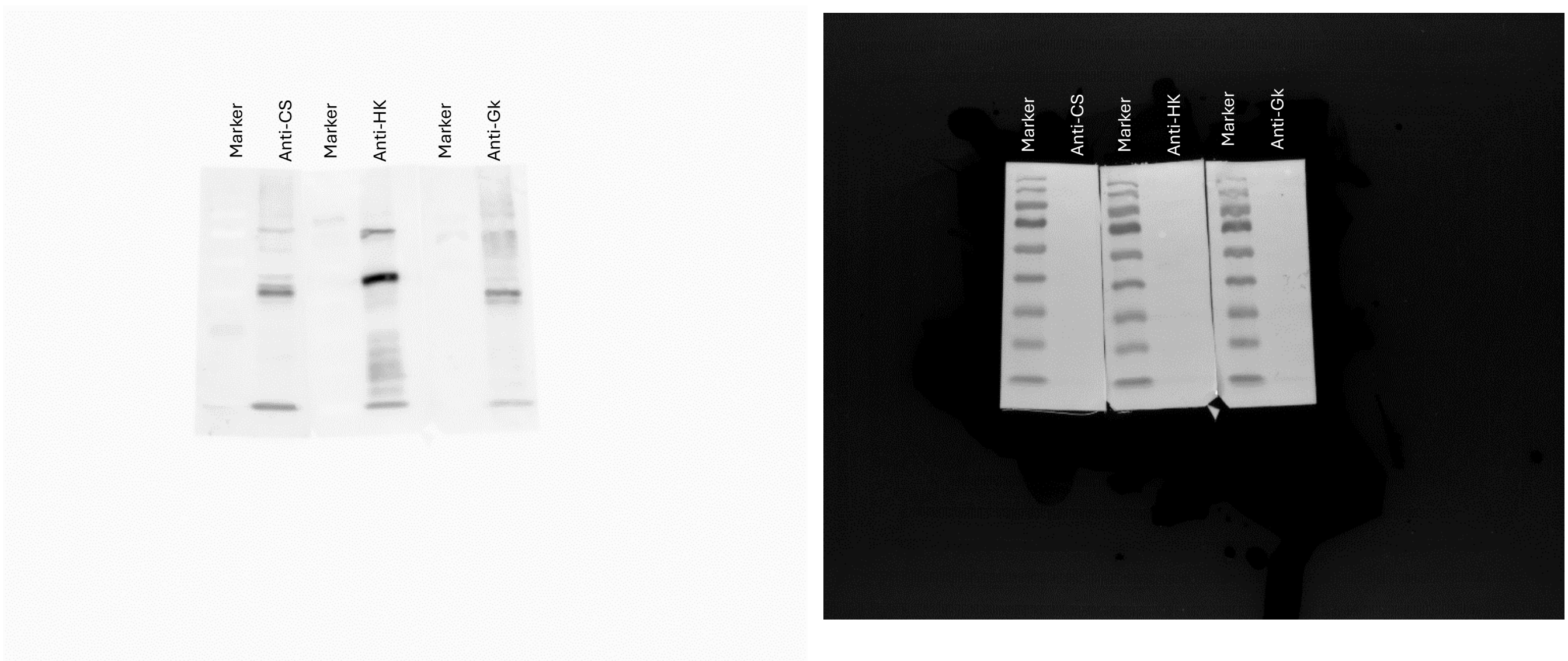

Supplement: Supplementary file 1 [file pntd.0013299.s016.pdf]
